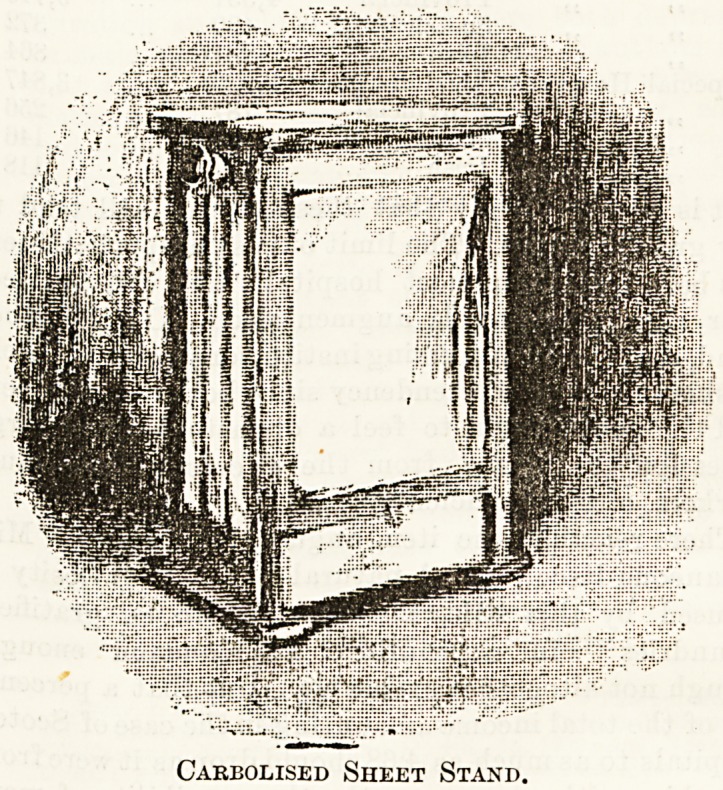# Practical Departments

**Published:** 1894-04-28

**Authors:** 


### PRACTICAL DEPARTMENTS.

### CARBOLISED SHEETS.

Amongst the various precautions which are adopted for the purification and disinfection of the atmosphere in the immediate neighbourhood of any case of infectious illness must be mentioned the use of a sheet wrung out in carbolic or some other disinfecting solution. Where strict isolation of the sick room is necessary such a sheet should be hung over the door, and kept thoroughly wet, with a view to counteracting in some measure the poison germs with which the air of the sick room may be impregnated, and thus acting as a barrier between the infected and other parts of the house.

Of course, this precaution is absolutely valueless unless the sheet be so arranged as entirely to cover the doorway and be kept in a state of continual moisture, and this latter accomplishment is not always an easy matter. Where possible it is well to use some contrivance such as Lacy's Isolation Sheet, which consists of a narrow zinc lined tank, easily secured with two brackets to the top of the doorway, and a perforated pipe which communicates with the tank and is attached to the sheet along the top. At one side is a tap, which being occasionally turned on will soak the sheet thoroughly. In this way the trouble of perpetually wringing out a heavy sheet with the hands, which seems to be the usual method, would be abolished. There does not, however, appear to us to be any occasion for this at any time, as given a sheet (or two sheets, which perhaps

Carbolised Sheet Stand.

are better, retaining the moisture for a longer period), attached say by three loops to three nails over the door, the use of an ordinary garden syringe, an easily procurable article, every now and then, would answer the purpose quite as effectively, and equally obviate the difficulty.

Keeping one of the lower corners in a small pot of water also is a good plan. With this help it is possible for the sheet to be kept thoroughly moist for twelve hours or so, and renders a regular re-soaking only an occasional necessity.

As deodorisers in hospital wards, stands such as the one here illustrated are most useful, and the plan upon which they are constructed is simple and good. A zinc or china tray filled with the disinfectant fits into the bottom of the stand, through which runs a roller. A corresponding roller is fitted at the top, and over these is placed a round towel. Thus it will be seen the lower part of the towel is perpetually in the solution, and a turn of a small handle at one side, communicating with the topmost roller, sends the towel round, wetting the whole of it thoroughly.

In wards where the sister or nurses have a predilection for Aspinall, these stands are quite attractive-looking, and with their marble or tiled tops are very convenient for the pots of ferns and flowers which it is so pleasant to see are now generally so abundant in our hospitals.

## Figures and Tables

**Figure f1:**